# TDD-YOLO: A novel model for precise detection of tomato diseases

**DOI:** 10.1371/journal.pone.0334989

**Published:** 2026-05-22

**Authors:** Zijian Chen, Zhihua Bian, Li Li, Chenxu Dai, Zhanlin Ji, Ivan Ganchev

**Affiliations:** 1 College of Mathematics and Computer Science, Zhejiang A&F University, Hangzhou, China; 2 Department of Information Engineering, Hebei University of Environmental Engineering, Qinhuangdao, China; 3 College of Artificial Intelligence, North China University of Science and Technology, Tangshan, China; 4 TRC/ECE, University of Limerick, Limerick, Ireland; 5 Faculty of Mathematics and Informatics, University of Plovdiv “Paisii Hilendarski”, Plovdiv, Bulgaria; 6 Institute of Mathematics and Informatics—Bulgarian Academy of Sciences, Sofia, Bulgaria; The University of British Columbia, AUSTRALIA

## Abstract

Tomato diseases pose a significant threat to global agricultural production, often leading to substantial yield loss and major economic damage. Traditional disease detection methods rely on manual inspection, which is not only time-consuming and labor-intensive but also difficult to implement for real-time monitoring. While deep learning-based object detection techniques offer a potential alternative to manual inspection, existing models still face challenges in extracting subtle disease features, suppressing complex background interference, and in handling multi-scale disease representations in complex agricultural environments, limiting detection performance. To address these limitations, this paper proposes a novel TDD-YOLO model for precise tomato-disease detection (TDD) in complex agricultural settings. The proposed model is based on YOLOv11 with the following three main improvements: (1) a feature enhancement module is added to improve the backbone’s ability to extract disease spot textures; (2) a joint attention mechanism is introduced to explicitly model cross-dimensional dependencies, effectively suppressing background interference; and (3) a feature fusion module is added to retain disease information across different scales while reducing computational costs. Experimental results, obtained on the Tomato-Village dataset (containing field-acquired images of tomato leaves with six diseases, collected in real agricultural environments, featuring complex backgrounds and varying illumination conditions) and Tomato-Disease dataset (emphasizing a greater diversity in tomato disease types along with healthy leaf samples), demonstrate that the proposed TDD-YOLO model outperforms the baseline in detection of tomato diseases (e.g., by improving mAP@50 and mAP@50:95, averaged across disease categories, by 4.1% and 6.0% on Tomato-Village and by 3.6% and 3.9% on Tomato-Disease, respectively) and state-of-the-art models (e.g., by improving the average mAP@50 and mAP@50:95, compared to the first runner-up, by 3.2% and 4.7% on Tomato-Village and by 2.4% and 2.1% on Tomato-Disease, respectively), while maintaining good parameter count and computational complexity, confirming its effectiveness and potential for practical usage in complex agricultural environments. The author-generated code and weight files are publicly available at https://github.com/LingShaQ/TDD-YOLOCode.

## Introduction

Tomato is one of the most important economic crops worldwide. It is widely cultivated due to its pleasant flavor and high nutritional value [1]. However, influenced by environmental and climatic factors, tomato plants are highly susceptible to infections caused by bacteria and viruses during their growth, which leads to yield reduction and quality degradation, and results in substantial economic losses for growers [2]. Traditional disease detection still relies mainly on manual field inspection or visual assessment, which is characterized by low efficiency, strong subjectivity, and limited spatial coverage, making it difficult to meet the requirements of large-scale and high-efficiency modern agricultural production [3]. Moreover, manual inspection is constrained by time and space, and it is often difficult to capture early leaf disease symptoms in a timely manner. This delay in diagnosis increases the risk of disease spread and further exacerbates yield loss [4].

In recent years, deep learning has achieved remarkable progress in crop disease detection and has become a key enabling technology for precision agriculture [5,6]. Among various models, the You Only Look Once (YOLO) family [7] of detectors has attracted considerable attention due to its real-time capability and high detection performance. As such, it has been widely applied for disease identification and fruit recognition in agricultural scenarios.

To address the challenge of detecting irregular diseases, many studies have attempted to combine object detection models with feature enhancement modules to improve the perception of small-scale lesions. Wang Q. et al. [8] proposed BED-YOLO—a tomato-disease detection model, based on YOLOv10n—by replacing the conventional convolution in the YOLOv10n’s backbone with a Deformable Convolutional Network (DCN) to adaptively perceive irregular lesions, significantly improving the detection sensitivity and localization accuracy of small disease regions, and achieving significant performance improvements compared to the original model. Abulizi et al. [9] introduced a tomato-disease detection model, named DM-YOLO, based on YOLOv9, by utilizing a lightweight dynamic upsampling DySample technique to enhance the feature extraction capability w.r.t. small lesions and suppress the interference from the background environment, and applying the MPDIoU loss function to enhance the learning of details of overlapping lesion margins. Li et al. [10] extended the receptive field by using Spatial-to-Depth Convolution (SPDConv), improving the YOLOv5’s ability to capture features of small rice lesions. Chen D. et al. [11] proposed the YOLOv8-MDN-Tiny model by replacing the YOLOv8’s backbone with their own More Focus on Small Objects (MFSO) structure to expand the feature pixels of small target information and enrich their feature expression, and proposed an improved DyRep fusion module to achieve precise localization of small-scale passion fruit diseases. Although these models alleviate the issue of missed detection to some extent, they mainly rely on traditional convolutional layers for enhancement, which limits their ability to extract edge detail features, making it difficult to capture the intricate details of irregular lesions.

Agricultural images are often interfered with by factors such as occlusion from branches and leaves, changes in lighting and shadows, and overlapping targets, making it difficult to accurately identify disease regions. To address this, some studies have introduced attention mechanisms to enhance the localization ability of respective models. For instance, Chen S. et al. [12] proposed the YOLO-COF model, which combines coordinate attention and K-means++ clustering to improve feature focusing ability in overlapping Camellia fruit scenes. Du et al. [13] designed the DSW-YOLO model, based on YOLOv7 with an additional DCN-ELAN module and shuffle attention, enabling it to effectively address the issue of occlusion by strawberry stems and leaves. Appe et al. [14] improved the YOLOv5 model by incorporating the Convolutional Block Attention Module (CBAM) [15], improving the focus on overlapping fruit features and achieving a certain accuracy. Although previous works have introduced modules such as CBAM to combine channel and spatial information for enhanced feature representation, the serial processing approach may lead to a neglect of certain disease features. This approach does not fully consider the dependencies between different dimensions, resulting in limitations when dealing with agricultural challenges w.r.t. weak disease textures and strong background interference, thus restricting the overall detection performance.

Due to significant scale differences in disease morphology, many researchers have combined feature fusion techniques with object detection methods to improve disease detection performance. For instance, Wang X. et al. [16] proposed the TomatoGuard-YOLO model, based on YOLOv10, by introducing a Multi-Path Inverted Residual Unit (MPIRU) to enhance multi-scale feature extraction and fusion, along with a dynamic focal attention framework (DFAF), substantially improving the tomato-disease detection performance. Lin et al. [17] used the BiFPN feature fusion network to design a tea-disease detection model, TSBA-YOLO, which addresses the challenge of identifying small-target tea leaf diseases. Chai et al. [18] proposed a dual-channel cross-feature fusion to improve YOLOv8n’s detection ability across different scales w.r.t. cherry tomato bunches. Wei et al. [19] constructed a feature refinement module (FRM) to improve the feature expression ability of YOLOv11, along with a SPPFELAN module to extract features of different levels and perform splicing and fusion, and proposed the GFS-YOLO11 model, addressing issues such as large-scale fruit differences, occlusion, and overlapping, existing in complex tomato field environments. Although these models perform well in the feature fusion modeling process, most of these fusion strategies employ fixed weights or serial processing mechanisms, which struggle to adaptively adjust the importance of features based on lesion scale and context. As a result, sensitivity to small targets is insufficient, or boundaries of large targets are misaligned, affecting the localization accuracy and convergence efficiency of respective models.

Although the C3k2 module in YOLOv11n improves inference efficiency and preserves spatial details, it exhibits insufficient capability in extracting fine-grained lesion textures within the backbone network [20]. Meanwhile, while the YOLOv11n’s C2PSA module introduces self-attention to capture long-range dependencies, it primarily focuses on global features and fails to model cross-dimensional interactions effectively [21]. To address the aforementioned issues, in this paper, a novel object detection TDD-YOLO model is proposed, based on YOLOv11n with the following enhancements, constituting the main contributions of this study:

1. To address the YOLOv11n backbone network’s insufficient ability to extract fine-grained disease-related textures, a newly designed Coordinate Attention Feature Enhancement (CAFE) module is proposed for utilization by the proposed TDD-YOLO model. Unlike conventional convolutions that employ a square receptive field, this module effectively uses local contextual information by applying asymmetric convolutions to model local context separately along the horizontal and vertical directions, making it more flexible and efficient when processing lesion regions, thereby enhancing the capture of fine-grained details and improving the local perception ability of TDD-YOLO.
2. To further improve the proposed model’s performance in detecting disease regions, a novel Channel-Spatial Attention Fusion (CSAF) module is introduced, which learns dependencies between channel and spatial dimensions through parallel interactive branches, thereby reducing background interference more effectively, and also strengthening the model’s focus on disease features. Most existing attention modules apply channel attention and spatial attention in a serial or parallel manner, whereas CSAF jointly models channel–spatial dependencies in parallel. This design avoids the order dependency of conventional serial structures and the feature isolation problem of typical parallel structures.
3. In response to low performance of the YOLOv11n’s neck in detecting tomato diseases due to single-scale fusion, a newly designed Multi-Scale Feature Fusion (MSFF) module is introduced, which adopts a multi-branch structure to fuse shallow detailed features with deep semantic features, thereby improving the proposed model’s detection performance while maintaining a low computational cost.

The performance of the proposed TDD-YOLO model is evaluated experimentally in comparison with the baseline model (YOLOv11n) and state-of-the-art models by utilizing two datasets. The obtained results demonstrate the superiority of TDD-YOLO.

## Materials and methods

### Datasets

In real production environments, tomato diseases are highly variable in form and appearance, with complex backgrounds. Therefore, this study utilized two different tomato-disease datasets to evaluate the performance of the proposed TDD-YOLO model in comparison to the baseline (YOLOv11n) and state-of-the-art models.

**Fig 1:**
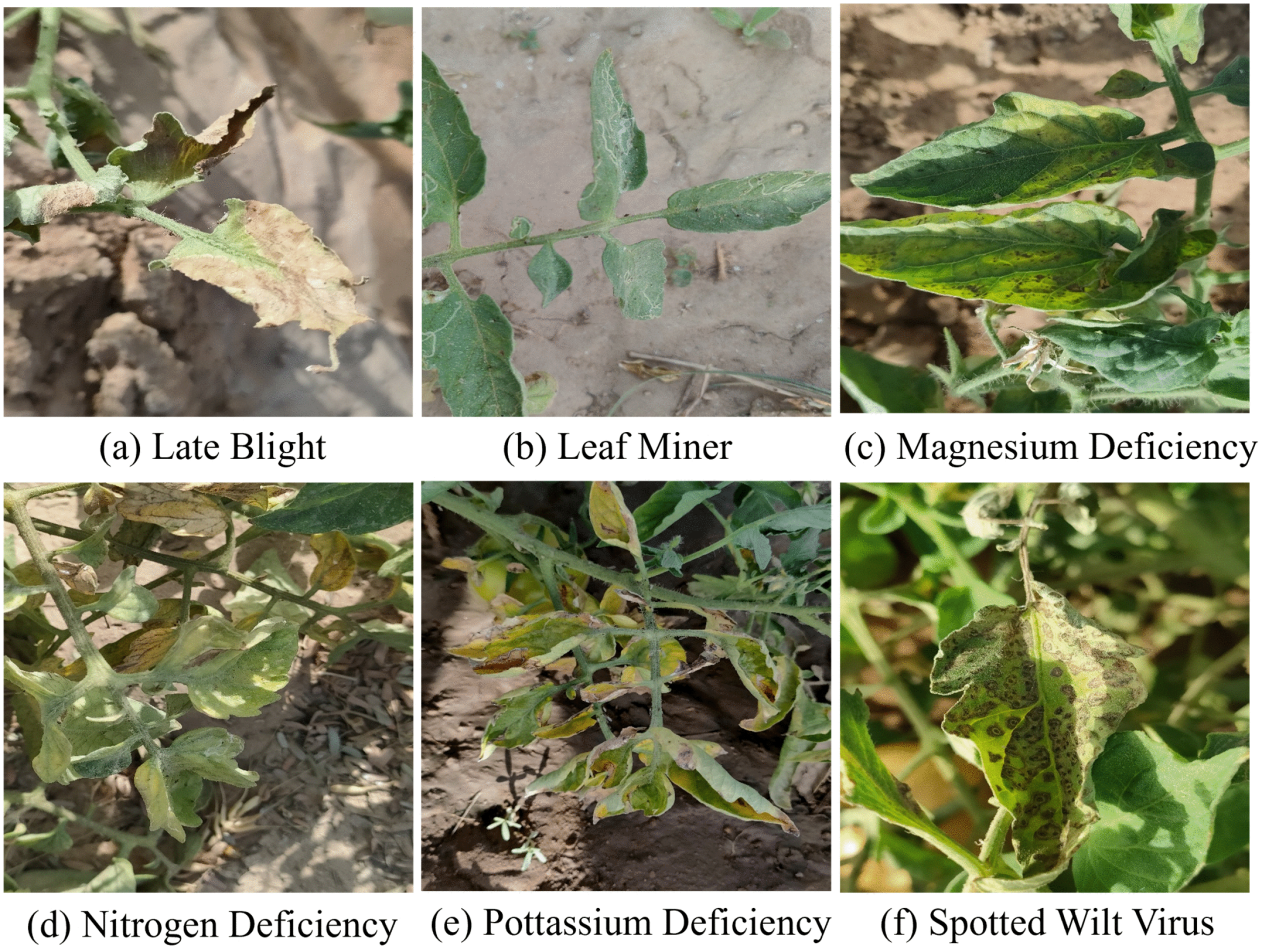
Sample images of the Tomato-Village dataset.

The first utilized dataset, namely Tomato-Village, was collected by Mamta Gehlot et al. [22] in the Jodhpur and Jaipur regions of Rajasthan (India), comprising 14,368 images of tomato leaves (containing 161,223 disease instances in total), captured under different growth stages, times, and illumination conditions. The images annotations were completed by Mamta Gehlot et al. with the aid of plant pathology literature, online resources, and guidance from local agricultural experts. The Tomato-Village dataset includes tomato leave images with six types of diseases, namely Late Blight, Leaf Miner, Magnesium Deficiency, Nitrogen Deficiency, Potassium Deficiency, and Spotted Wilt Virus, as illustrated in Fig 1. The diversity of diseases and backgrounds in this dataset more realistically reflects the challenges of disease detection in field scenarios and helps improve generalization ability of detection models.

The second utilized dataset, namely Tomato-Disease [23], was constructed using tomato leave images collected in Handan City (Hebei Province, China), as well as from online sources. The data collection period spanned from April to late August 2023, with images captured at 8:00 a.m. and 5:00 p.m., saved in JPEG format. The acquisition devices included an OPPO A5 smartphone and a Canon EOS 1500D camera. The photographs were captured under natural-field conditions, including realistic agricultural backgrounds such as soil and sky. To enhance dataset diversity and improve model generalization, images were collected under varying illumination conditions and backgrounds, with resolutions ranging from 143 × 137 to 8000 × 6000.

**Fig 2:**
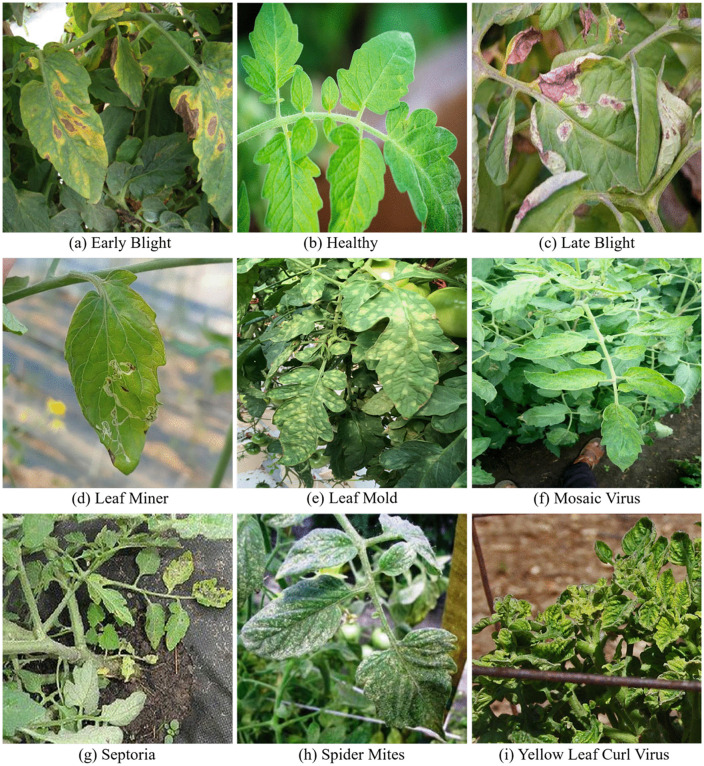
Sample images of the Tomato-Disease dataset.

The Tomato-Disease dataset contains 2212 images of tomato leaves, either healthy or affected by various diseases (with 6585 disease instances in total), featuring different sizes, shapes, and backgrounds. The covered tomato diseases include Early Blight, Late Blight, Leaf Miner, Leaf Mold, Mosaic Virus, Septoria, Spider Mites, and Yellow Leaf Curl Virus. The disease categories were confirmed under expert diagnostic guidance. All images were manually annotated using the LabelImg tool (v1.8.6). Disease regions were labeled using the largest possible horizontal bounding rectangles to fully cover visible lesions. The annotations were saved in YOLO-format TXT files and COCO-compatible JSON files to facilitate training and evaluation across different frameworks. To prevent overfitting and enhance generalization, online data augmentation strategies were applied during training, including random horizontal flipping (with probability of 0.5) and random vertical flipping (with probability of 0.2), and all training images were resized to 640 × 640 pixels. Finally, pixel values were normalized using the dataset mean and standard deviation, and the processed images together with the corresponding annotation files formed the standardized dataset used for model training and validation. Sample images of the Tomato-Disease dataset are shown in Fig 2.

Notably, the two utilized datasets share only two overlapping disease categories (Late Blight and Leaf Miner), while the remaining categories are non-overlapping, thereby covering a broader range of diseases for model performance evaluation.

To ensure effective model training and evaluation, since a single image may contain multiple disease instances, we adopted a stratified data-splitting strategy based on annotated instances. Each dataset was divided into training, test, and validation subsets at a ratio of 8:1:1 using stratified random sampling. This division provided a sufficient number of samples for model training while retaining enough data for accurate assessment of the performance. Tables 1 and 2 report the distribution of annotated instances of each disease category in the Tomato-Village dataset and Tomato-Disease dataset, respectively.

**Table 1:** Distribution of annotated disease instances in the Tomato-Village dataset.

Disease Category	Training Set	Validation Set	Test Set	Total
**Late Blight**	3174	398	366	**3938**
**Leaf Miner**	85209	11130	10860	**107199**
**Magnesium Deficiency**	12503	1611	1756	**15870**
**Nitrogen Deficiency**	1310	182	201	**1693**
**Potassium Deficiency**	869	114	143	**1126**
**Spotted Wilt Virus**	25252	3148	2997	**31397**
**Total**	**128317**	**16583**	**16323**	**161223**

**Table 2:** Distribution of annotated disease instances in the Tomato-Disease dataset.

Disease Category	Training Set	Validation Set	Test Set	Total
**Early Blight**	597	78	107	**782**
**Healthy**	607	71	58	**736**
**Late Blight**	354	68	51	**473**
**Leaf Miner**	681	67	88	**836**
**Leaf Mold**	635	91	69	**795**
**Mosaic Virus**	504	86	64	**654**
**Septoria**	492	49	57	**598**
**Spider Mites**	376	71	51	**498**
**Yellow Leaf Curl Virus**	984	107	122	**1213**
**Total**	**5230**	**688**	**667**	**6585**

**Fig 3:**
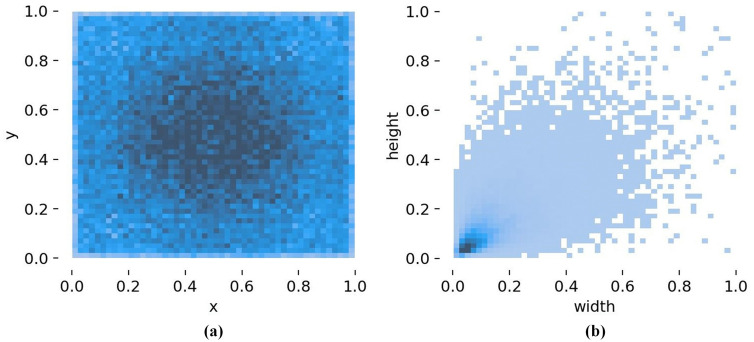
Histogram visualization charts of the Tomato-Village dataset’s annotation file statistics: (a) spatial distribution of target center points; (b) distribution of disease bounding-box widths and heights.

**Fig 4:**
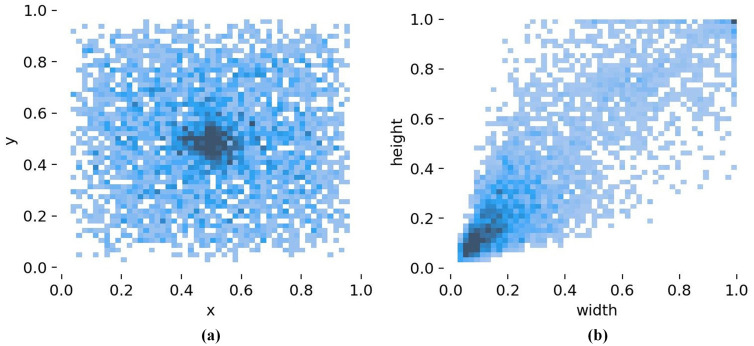
Histogram visualization charts of the Tomato-Disease dataset’s annotation file statistics: (a) spatial distribution of target center points; (b) distribution of disease bounding-box widths and heights.

Figs 3 and 4 present the histogram visualization charts of annotation files of the Tomato-Village dataset and Tomato-Disease dataset, respectively.

Specifically, Fig 3a illustrates the spatial distribution of target center points, which is used to analyze the positional characteristics of diseases within the images. It can be observed that, in the Tomato-Village dataset, disease instances are distributed across the entire image area, while the darker color in the central region indicates a higher concentration of instances near the image center. Fig 3b depicts the distribution of disease bounding-box widths and heights, which is employed to analyze disease scale characteristics and variations in aspect ratios, providing a relatively detailed statistical description of disease scales. As shown in Fig 3b, the density is higher in regions where the width and height are less than 0.15, indicating that the number of small-size targets is significantly greater than that of large targets. The distribution of disease instances exhibits a fan-shaped pattern, suggesting substantial diversity and differences in disease shapes, which places higher requirements on the model’s ability to extract small-target features and to adapt to scale variations.

As shown in Fig 4a, the darker color in the central region indicates that disease targets in the Tomato-Disease dataset are mainly concentrated near the image center. Fig 4b shows that the highest density occurs in the region where the width and height are less than 0.2, indicating that this dataset contains more small targets than large ones. Moreover, the disease distribution exhibits a clear trend along the diagonal direction, demonstrating that the disease shapes are close to a square proportion, which imposes higher requirements on the model’s feature extraction capability.

### Proposed model: TDD-YOLO

The proposed TDD-YOLO model is based on the single-stage object detection YOLOv11n model [24], which inherits the efficiency and real-time performance of the YOLO series, while introducing further optimizations and innovations. YOLOv11n consists of three main parts: a backbone, a neck, and a head. The backbone employs kernel-2 Cross Stage Partial (C3k2), Spatial Pyramid Pooling Fast (SPPF), and Cross-Stage Partial + Spatial Attention (C2PSA) modules to enhance feature extraction capabilities. The neck, located between the backbone and the head, is responsible for feature fusion and enhancement. The head is the decision-making part, responsible for generating the final detection results.

YOLOv11n incorporates lightweight modules, such as C3k2 and C2PSA, which significantly reduce its parameter count and floating-point operations per second (FLOPs). This design leads to faster inference speed, enabling the model to meet real-time object detection requirements. Compared with other YOLO variants, YOLOv11n introduces a more streamlined architecture and an optimized training pipeline, achieving better balance between detection performance and efficiency with fewer parameters and lower computational complexity. Moreover, by employing optimization techniques, such as depthwise separable convolution, the overall model’s size is further reduced, making YOLOv11n highly suitable for deployment in agricultural environments on mobile and edge devices with limited computational resources. Therefore, YOLOv11n was selected as a baseline model in this study.

**Fig 5:**
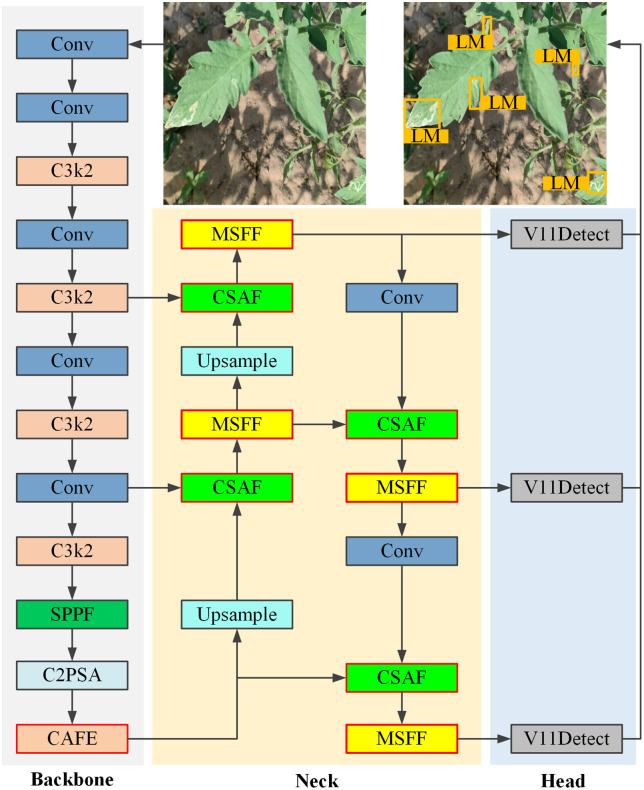
The structure of the proposed TDD-YOLO model (the newly designed modules are marked with red contour; Leaf Miner (LM) is one of the tomato diseases targeted in this study).

However, even though YOLOv11n demonstrates good real-time performance and efficient feature extraction, it still faces challenges in tomato-disease detection. These include insufficient extraction of low-level features and limited capability in handling multi-scale disease features. To address these issues, we designed a new model, TDD-YOLO (Fig 5), for improved tomato-disease detection. TDD-YOLO is based on YOLOv11n by incorporating three newly designed module types into it, namely CAFE, CSAF, and MSFF. These are described in detail in the following subsections.

### CAFE module

In real-world tomato-disease images, early lesions typically appear as slight discoloration or tiny lesions similar to the leaf background. YOLOv11n primarily relies on the backbone to extract high-dimensional features. While the YOLOv11n backbone offers high speed and efficiency, it tends to lose fine-grained local textures during deep feature extraction due to its limited capacity for low-level feature representation. This degradation of detail-rich disease features often leads to missed detections, particularly in complex agricultural scenes.

**Fig 6:**
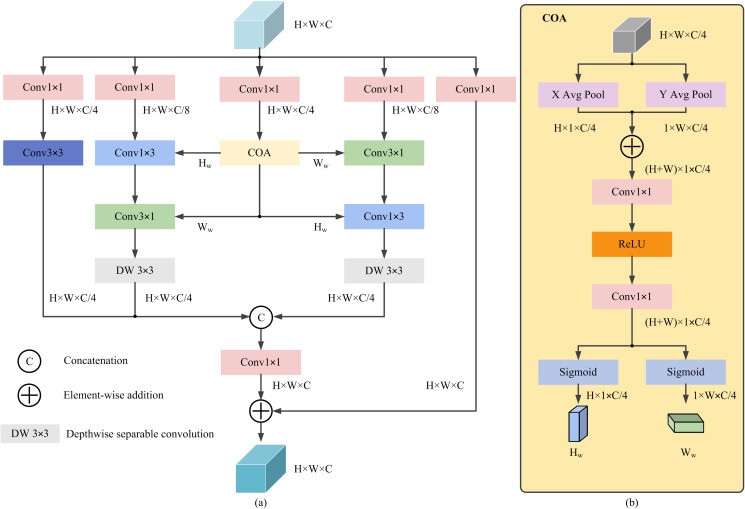
The newly designed CAFE module, utilized by the proposed TDD-YOLO model: (a) the overall CAFE structure; (b) the COA submodule used by it.

To address this issue, a novel CAFE module, based on an improved coordinate attention mechanism [25] is proposed here for incorporation into YOLOv11n (after the C2PSA block in the backbone) for enhanced feature fusion. Given an input feature map $x\in {\text{R}}^{H\times W\times C}$, where C denotes the number of channels and H×W represents the spatial resolution, the CAFE module produces an output ${CAFE}_{out}\in {\text{R}}^{H\times W\times C}$ with identical dimensions. By integrating attention guidance with asymmetric convolution, CAFE effectively fuses low-level and high-level features, capable of extracting local detail features, enriching feature representation, and improving both the localization accuracy and detection performance for diseased regions. The structure of the CAFE module is depicted on Fig 6a.

The input features first pass through five parallel 1 × 1 convolutions to extract spatial information, forming the beginning of five CAFE branches. The first and third branches each generate C/4 channel features, with the first branch being used to retain the feature information of minor defects. The third branch then uses a Coordinate Attention (COA) submodule (Fig 6b), which extracts spatial information in different directions by applying average pooling separately along the horizontal and vertical axes, effectively capturing global contextual information. A multi-layer perceptron (MLP) is then used by COA to generate spatial attention vectors, which helps the proposed model better understand spatial dependencies within images. Finally, a sigmoid function is applied to produce horizontal direction weights $H_{w}$ and vertical direction weights $W_{w}$, with spatial sizes of H×1×C/4 and 1×W×C/4, respectively, thereby preserving the shape characteristics and spatial distribution of disease regions on the leaf surface. This can be expressed as follows:


$$\begin{array}{c} H_{w}=\sigma \left(MLP\left({AvgPool}_{h}(x)\right)\right) \end{array}$$ (1)

$$\begin{array}{c} W_{w}=\sigma \left(MLP\left(Avg{Pool}_{w}(x)\right)\right) \end{array}$$ (2)

where x denotes the input feature map, AvgPool denotes an average pooling, and σ denotes the sigmoid function. The extracted features are dynamically weighted and adjusted by the COA submodule to emphasize critical information.

The obtained $H_{w}$ and $W_{w}$ are then used by the second and fourth CAFE branches, which first apply a 1 × 1 convolution for channel alignment to ensure dimensional consistency for the subsequent element-wise multiplication, obtaining features with a spatial size of H×W×C/4, and then apply asymmetric convolutions in different orders to extract features along horizontal and vertical directions. A 3 × 3 depthwise separable convolution is then used to expand the receptive field with fewer computations and parameters, allowing more contextual information to be retained while improving the model efficiency and performance.

Meanwhile, the first (leftmost) branch uses a 3 × 3 convolution to extract local fine-grained features of diseases, thereby enhancing the ability of the proposed model to understand subtle pathological details. The fifth (rightmost) branch adopts a residual structure to preserve essential features of small-scale lesions.

Finally, the feature maps output by the first, second, and fourth branches are concatenated along the channel dimension, followed by a 1 × 1 convolution and an element-wise addition with the output of the fifth branch to produce the CAFE output.

The operation of the CAFE module can be expressed as follows:


$$\begin{array}{c} \;F_{\text{1}}=Conv\text{2}d_{\text{1}\times \text{1}}\left(Cat\left\{ \begin{array}{cc} \;\;\;\;\;\;\;\;\;\;\;\;\;\;\;\;\;\;\;\;\;\;\;\;\;\;\;\;\;\;\;\;\;\;\;\;\;\;\;\;\;\;\;\;\;\;\;\;\;\;\;\;\;\;\;\;Conv\text{2}d_{\text{3}\times \text{3}}\left(Conv\text{2}d_{\text{1}\times \text{1}}(x)\right) & \\ DW_{\text{3}\times \text{3}}\left(Conv\text{2}d_{\text{3}\times \text{1}}\left(Conv\text{2}d_{\text{1}\times \text{3}}(x)⊙H_{w}\right)⊙W_{w}\right) & \end{array}\right.\;\;\;\;\;\right)) \end{array}$$ (3)

$$\begin{array}{c} {CAFE}_{out}=ReLU\left(F_{\text{1}}\right)+Conv\text{2}d_{\text{1}\times \text{1}}(x) \end{array}$$ (4)

where $DW_{\text{3}\times \text{3}}$ denotes a depthwise separable convolution, Cat denotes a concatenation operation, ⊙ denotes an element-wise multiplication, and ${CAFE}_{out}$ denotes the final output of the CAFE module.

Results, obtained by ablation study experiments further presented in this paper, showed that introducing the CAFE module into the backbone increases both parameter count from 2.59M to 2.90M and FLOPs from 6.3G to 6.6G (c.f., Table 8), but enables the proposed model to better capture disease features, improves its detection performance in complex backgrounds, and enhances its accuracy.

### CSAF module

In complex agricultural environments, tomato-disease regions often share many similarities with the surrounding environment, causing a model to be easily affected by background interference during disease detection. Therefore, attention mechanisms are commonly introduced to enhance the model focus on disease-specific features while suppressing irrelevant background information. For example, CBAM [15] first adjusts channel importance through channel attention, and then adjusts spatial importance through spatial attention, thereby improving the feature representation capability. However, this serial processing method may lead to the omission of important information in earlier stages, as it overlooks the interdependencies among different dimensions. This limitation affects the overall model performance.

**Fig 7:**
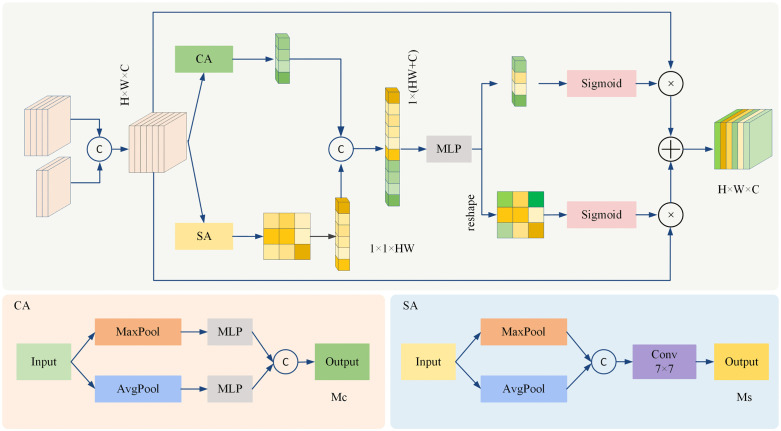
The newly designed CSAF module, utilized by the proposed TDD-YOLO model.

Although YOLOv11n integrates a self-attention mechanism, it often fails to fully utilize the spatial dimension information of the features, and focuses more on capturing global information, ignoring the importance of local details. To address this issue, a new attention mechanism and corresponding module, called CSAF, is proposed in this paper. The CSAF module, depicted in Fig 7, is utilized in the fusion nodes of different feature connections within the neck. The objective of this module is to further enhance the ability of the proposed model to focus on key disease features, especially in situations where the background is complex and the disease area is similar to the background. By dynamically integrating channel and spatial attention, the corresponding CSAF module not only enhances the proposed model’s focus on key disease features but also captures complex dependencies across different dimensions in the feature maps.

The CSAF module consists of two branches. The upper branch (c.f., Fig 7) utilizes max pooling and average pooling to extract global contextual information from the input feature $x\in {\text{R}}^{H\times W\times C}$, capturing the importance of each channel in the feature map as well as inter-channel dependencies. MLP is then used to generate a nonlinear map, assigning weight values to each channel and producing channel attention weights $M_{c}$ with a spatial size of 1×1×C. The lower branch applies max pooling and average pooling along the channel dimension of the input feature map to capture both the prominent lesion regions and overall distribution information. This step enables the proposed model to better understand the spatial location and relative size of disease features in images. Subsequently, a 7 × 7 convolution is applied to generate spatial attention weights $M_{S}$ with a spatial size of H×W×1.

The operation of the CSAF module can be expressed as follows:


$$\begin{array}{c} M_{c}(x)=\sigma \left(Cat\left(MLP\left(AvgPool(x)\right),MLP\left(MaxPool(x)\right)\right)\right) \end{array}$$ (5)

$$\begin{array}{c} M_{s}(x)=\sigma \left({Conv}_{\text{7}\times \text{7}}\left(Cat\left(AvgPool(x),MaxPool(x)\right)\right)\right) \end{array}$$ (6)

To further enhance the expressive capability of the CSAF module, $M_{S}$ is flattened along the channel dimension to obtain a feature of size 1×1×HW and is concatenated with $M_{c}$ to form a new joint feature representation, which dynamically adjusts the number of feature channels. Two 1 × 1 convolutions are then used to fuse the global spatial features and channel features across modalities, thus modeling the nonlinear dependencies between the two types of attentions. The fused features are then split back into the original channel and spatial dimensions, followed by normalization using a sigmoid function to maintain the stability of the attention mechanism. After information fusion across different dimensions, the channel and spatial weights are element-wise multiplied with the input feature map and then summed to generate the output with a spatial size of H×W×C.

By introducing the newly designed CSAF modules into the neck, parameter count increases from 2.59M to 2.68M and FLOPs increases from 6.3G to 6.5G (c.f., Table 8), but the ability of the proposed TDD-YOLO model to focus on critical disease features is improved by dynamically adjusting channel- and spatial information. This way, TDD-YOLO can effectively capture both the local detail features of leaf lesions and their global contextual relationships.

**Table 8:** Ablation study results, obtained on the Tomato-Village dataset (the best values are bolded).

Step	MSFF	CSAF	CAFE	Average Precision	Ave rage Recall	Ave rage mAP@50	Ave rage mAP@50:95	Parameter count (M)	FLOPs (G)	FPS
0	×	×	×	0.859	0.782	0.838	0.546	2.59	6.3	**63.79**
1	✓	×	×	0.876	0.773	0.852	0.556	**2.17**	**6.1**	58.47
2	×	✓	×	0.875	0.790	0.851	0.553	2.68	6.5	35.47
3	×	×	✓	0.879	0.791	0.855	0.564	2.90	6.6	60.23
4	✓	✓	×	0.876	0.779	0.856	0.555	2.27	6.2	53.41
5	✓	×	✓	0.842	0.803	0.859	0.567	2.48	6.3	57.43
6	×	✓	✓	0.878	0.794	0.858	0.570	3.00	6.7	32.87
7	✓	✓	✓	**0.886**	**0.805**	**0.872**	**0.579**	2.59	6.5	51.54

### MSFF module

**Fig 8:**
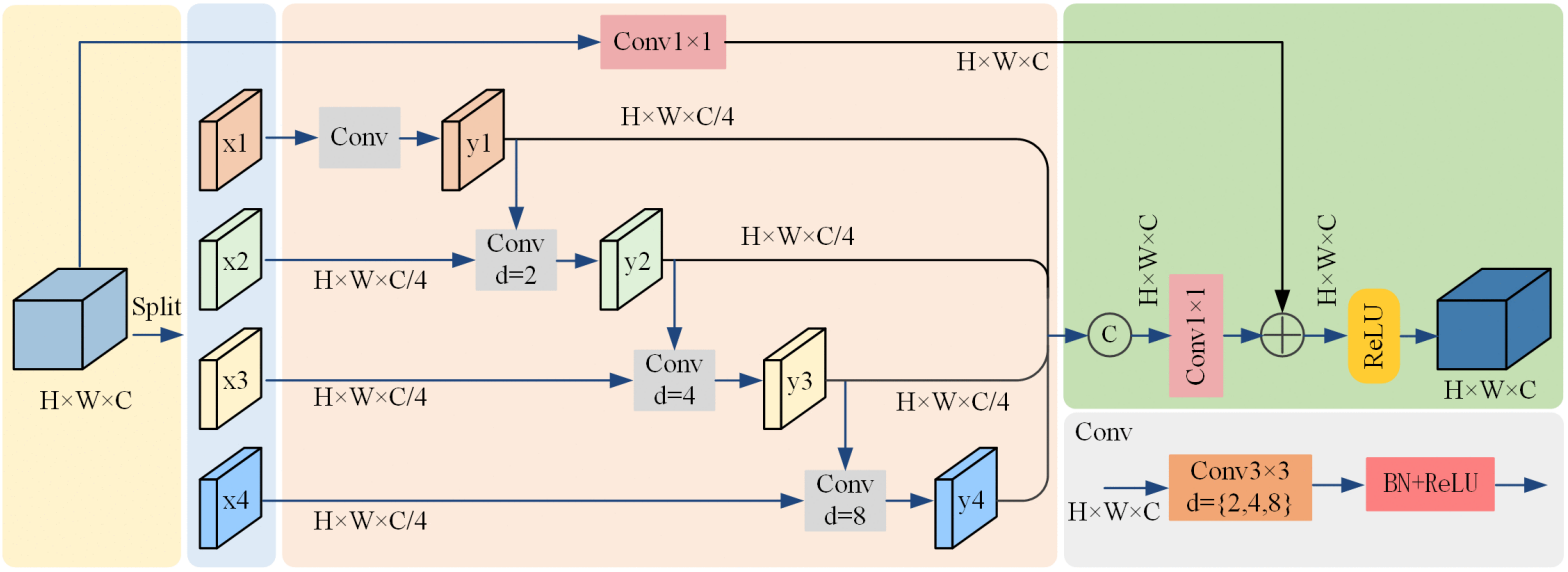
The newly designed MSFF module, utilized by the proposed TDD-YOLO model.

In the actual agricultural production environment, tomato diseases are complex and diverse, and disease features at different scales often appear mixed. Single-scale feature extraction methods struggle to accommodate this diversity and scale variation, leading to the potential loss of critical disease information and resulting in reduced detection performance. Although the C3k2 module enhances the feature extraction capability of YOLOv11n, it still struggles to capture both local details and global context simultaneously, when handling multi-scale disease features. This limitation affects the model’s robustness and generalization performance. Therefore, in order to address the limitations of single scale feature extraction methods in multi-scale disease feature recognition and address the problem of increased parameter count, caused by the introduction of CSAF and CAFE modules, a novel MSFF module was designed, as shown in Fig 8. A MSFF module replaces each C3k2 module in the neck of the proposed TDD-YOLO model. The MSFF module adopts a multi-branch structure to capture feature information under varying receptive fields, which addresses the limitations of single-scale feature extraction methods and improves the model’s ability of recognizing multi-scale disease features.

Given an input feature map $x\in {\text{R}}^{H\times W\times C}$, the MSFF module first evenly splits it into four sub-features {x1, x2, x3, x4} along the channel dimension, and then feeds each sub-feature into four separate branches for further processing. This approach reduces the model’s parameter count and allows each branch to focus more effectively on extracting information at specific scales. As local features often contain vital disease details like leaf spots or color changes, which are crucial for distinguishing the diseased areas, the first branch uses a standard 3 × 3 convolution to extract local texture features. The other three branches apply dilated convolutions with different dilation rates to gradually expand the receptive field and capture multi-scale contextual information, producing features $y_{i}\in {\text{R}}^{H\times W\times C/\text{4}}$. This design of the MSFF module allows the proposed TDD-YOLO model to capture more disease-related information, which helps enhance its ability to accurately locate disease boundaries and distinguish diseased regions from the background.

The outputs of the four branches are concatenated in the channel dimension. Then, a 1 × 1 convolution is used for feature fusion. This integration of multi-scale feature information helps the proposed model focus on local details and understand the global context. Additionally, to further improve training stability and convergence speed, residual connections and a scaling factor λ are introduced to enhance the gradient flow and alleviate the vanishing gradient problem in deep network layers, yielding an output with a spatial size of H×W×C. The operation of the MSFF module can be expressed as follows:


$$\begin{array}{c} y_{\text{1}}={Conv}_{\text{3}\times \text{3}}\left(x_{\text{1}}\right) \end{array}$$ (7)

$$\begin{array}{c} y_{i}={Conv}_{\text{3}\times \text{3}}\left(y_{i-\text{1}}+x_{i}\right) \end{array}$$ (8)

$$\begin{array}{c} output=\lambda \cdot {Conv}_{\text{1}\times \text{1}}\left(Cat\left\{y_{\text{1}}{,y}_{\text{2}},y_{\text{3}},y_{\text{4}}\right\}\right)+{Conv}_{\text{1}\times \text{1}}(x) \end{array}$$ (9)

where $x_{i}$ denotes the input feature of the i-th branch, $y_{i}$ denotes the output of the i-th branch, and output denotes the output feature map.

Experimental results, obtained by the ablation study further presented in this paper, demonstrate that the utilization of MSFF modules by the proposed model allows to reduce both the parameter count from 2.59M to 2.17M and FLOPs from 6.3G to 6.1G (c.f., Table 8). In addition, the multi-scale feature fusion capability of the MSFF module significantly contributes to the enhanced tomato-disease detection performance of the proposed model by improving its ability to differentiate between various disease types and lesion regions. Its module design effectively addresses the limitations of single-scale feature extraction methods, which often miss important disease characteristics. By integrating features across multiple scales, the MSFF modules improve the representation of key disease areas and strengthen the overall detection performance of the proposed TDD-YOLO model.

## Experiments and results

### Evaluation metrics

To evaluate the performance of the proposed TDD-YOLO model in comparison to the baseline and state-of-the-art models, the following metrics were used: precision, recall, mAP@50, mAP@50:95, and frame rate per second (FPS).

Precision (*P*) represents the ratio of true positive (TP) samples to all positive samples detected by a model, as shown in (10), where FP denotes the number of samples incorrectly detected as positive by a model. Recall (*R*) represents the ratio of TP samples to all actual positive samples, as shown in (11), where FN denotes the number of samples missed by a model. Average precision (AP) is represented as the integral of the precision–recall curve over different recall values, as shown in (12). In this study, the precision–recall curves are macro-averaged across all disease categories to ensure equal contribution from both common and rare categories. Mean average precision (mAP) is the average of the AP values across all categories used to comprehensively evaluate the overall performance of a model, as shown in (13), where N denotes the total number of categories and ${AP}^{\left(k\right)}@50$ denotes the AP of category k, measured using a fixed Intersection over Union (IoU) threshold of 0.50 (if the IoU between a predicted bounding box and the ground-truth bounding box is greater than or equal to 0.5, the detection is regarded as correct). mAP@50 evaluates models under relatively low localization requirements. In contrast, mAP@50:95 averages mAP calculated at 10 IoU thresholds, ranging from 0.50 to 0.95 in steps of 0.05, as shown in (14). Generally, mAP@50:95 provides a stricter and more comprehensive evaluation than mAP@50. Higher values of mAP@50:95 indicate better detection performance across different localization accuracy requirements. FPS represents the inference speed of a model, indicating how many frames it can process per second. It is a key metric for evaluating the real-time performance and efficiency of a model. A higher frame rate can ensure that a model can perform detection tasks quickly enough to meet real-time requirements. In the conducted experiments during inference, the confidence threshold was set to 0.25 and the IoU threshold for the Non-Maximal Suppression (NMS) was set to 0.70.


$$\begin{array}{c} P=\frac{TP}{TP+FP} \end{array}$$ (10)

$$\begin{array}{c} R=\frac{TP}{TP+FN} \end{array}$$ (11)

$$\begin{array}{c} AP=\int_{\text{0}}^{\text{1}}P(R)dR \end{array}$$ (12)

$$\begin{array}{c} mAP@\text{50}=\frac{\text{1}}{N}\sum_{k=\text{1}}^{N}AP^{\left(k\right)}@50 \end{array}$$ (13)

$$\begin{array}{c} mAP@\text{50}\;\;:\;\;\text{95}=\frac{\text{1}}{\text{1}\text{0}}(mAP@\text{5}\text{0}+mAP@\text{5}\text{5}+\ldots +mAP@\text{9}\text{5}) \end{array}$$ (14)

### Experimental environment and parameter settings

The experimental environment values and parameter settings (summarized in Table 3) were the same for both datasets used, except for the number of epochs which was set (based on the loss curves) to 500 for Tomato-Village and 200 for Tomato-Disease, which reflects the differences in their sizes and complexity. The Tomato-Village dataset contains more disease images and more complex disease patterns, and thus 500 epochs were required to ensure sufficient convergence. In contrast, the smaller Tomato-Disease dataset allows for faster convergence, while continued model training may lead to overfitting, so the number of epochs was set to 200. The training time for the proposed TDD-YOLO model on the Tomato-Village dataset was approximately 11.9 hours, while on the Tomato-Disease dataset, it was around 0.78 hours. The training time for the baseline (YOLOv11n) on the Tomato-Village dataset was approximately 13.1 hours, while on the Tomato-Disease dataset, it was around 0.83 hours. The SGD optimizer was used in the training process.

**Table 3:** Experimental environment and parameter settings.

Item/parameter	Description/value
CPU	Intel(R) Xeon(R) Silver 4214R CPU @ 2.40GHz
GPU	RTX 3080 Ti
Operating system	Linux
Cuda version	11.3
Python version	3.8
PyTorch version	1.10.0
Workers	8
Batch size	32
Resolution	640 × 640 pixels
Learning rate	0.01
Momentum coefficient	0.937
Decay coefficient	0.0005625
Seed	0
Number of independent experimental runs	10

### Performance comparison with baseline

First, we compared the proposed TDD-YOLO model with the baseline model (YOLOv11n) in the task of tomato-disease detection, performed on the Tomato-Village and Tomato-Disease datasets. The obtained experimental results are presented in Tables 4 and 5, respectively.

**Table 4:** Performance comparison with the baseline, performed on the Tomato-Village dataset (the better values are bolded).

Disease Category	YOLOv11n (*baseline*)				TDD-YOLO (*proposed*)			
	Precision	Recall	mAP@50	mAP@50:95	Precision	Recall	mAP@50	mAP@50:95
**Late** **Blight**	0.854 ±0.004	**0.857** ±0.001	0.893 ±0.003	0.583 ±0.002	**0.875** ±0.001	0.854 ±0.002	**0.904** ±0.003	**0.609** ±0.001
**Leaf** **Miner**	0.861 ±0.003	**0.656** ±0.002	0.765 ±0.003	0.451 ±0.001	**0.881** ±0.002	0.647 ±0.002	**0.768** ±0.001	**0.461** ±0.001
**Magnesium** **Deficiency**	0.887 ±0.001	0.823 ±0.003	0.885 ±0.002	0.604 ±0.002	**0.918** ±0.004	**0.841** ±0.003	**0.918** ±0.003	**0.621** ±0.002
**Nitrogen** **Deficiency**	**0.855** ±0.004	0.730 ±0.002	0.775 ±0.003	0.478 ±0.003	0.852 ±0.002	**0.796** ±0.002	**0.829** ±0.003	**0.523** ±0.002
**Potassium** **Deficiency**	0.807 ±0.003	0.847 ±0.001	0.857 ±0.003	0.621 ±0.002	**0.879** ±0.002	**0.910** ±0.003	**0.946** ±0.001	**0.693** ±0.002
**Spotted Wilt** **Virus**	0.892 ±0.003	0.775 ±0.004	0.851 ±0.003	0.538 ±0.002	**0.909** ±0.004	**0.781** ±0.001	**0.865** ±0.002	**0.568** ±0.001
***Average*** ***values***	*0.859* *±0.003*	*0.781* *±0.001*	*0.838* *±0.002*	*0.546* *±0.002*	***0.886*** *±0.003*	***0.805*** *±0.001*	***0.872*** *±0.002*	***0.579*** *±0.001*

**Table 5:** Performance comparison with the baseline, performed on the Tomato-Disease dataset (the better values are bolded).

Disease Category	YOLOv11n (*baseline*)				TDD-YOLO (*proposed*)			
	Precision	Recall	mAP@50	mAP@50:95	Precision	Recall	mAP@50	mAP@50:95
**Early** **Blight**	0.685 ±0.004	**0.751** ±0.003	0.715 ±0.001	0.541 ±0.002	**0.815** ±0.003	0.695 ±0.001	**0.787** ±0.001	**0.626** ±0.001
**Healthy**	0.543 ±0.002	**0.495** ±0.002	**0.626** ±0.002	0.493 ±0.002	**0.691** ±0.002	0.465 ±0.002	0.615 ±0.002	**0.496** ±0.002
**Late** **Blight**	0.723 ±0.003	0.790 ±0.003	**0.855** ±0.002	**0.671** ±0.001	**0.750** ±0.002	**0.818** ±0.002	0.842 ±0.001	0.658 ±0.005
**Leaf** **Miner**	0.826 ±0.004	**0.879** ±0.004	0.915 ±0.002	0.767 ±0.002	**0.872** ±0.002	0.825 ±0.002	**0.927** ±0.001	**0.768** ±0.001
**Leaf** **Mold**	0.629 ±0.003	**0.624** ±0.003	0.638 ±0.001	0.434 ±0.002	**0.720** ±0.002	0.577 ±0.004	**0.675** ±0.002	**0.460** ±0.003
**Mosaic** **Virus**	0.859 ±0.002	0.812 ±0.002	0.896 ±0.002	0.780 ±0.001	**0.926** ±0.002	**0.850** ±0.002	**0.945** ±0.001	**0.813** ±0.001
**Septoria**	0.612 ±0.003	0.389 ±0.002	0.519 ±0.002	0.318 ±0.002	**0.754** ±0.002	**0.401** ±0.002	**0.623** ±0.002	**0.419** ±0.006
**Spider** **Mites**	0.889 ±0.004	**0.918** ±0.001	**0.943** ±0.001	**0.883** ±0.002	**0.955** ±0.003	0.917 ±0.002	0.934 ±0.001	0.868 ±0.001
**Yellow Leaf Curl** **Virus**	0.737 ±0.003	**0.727** ±0.003	0.810 ±0.003	**0.485** ±0.002	**0.913** ±0.002	0.695 ±0.003	**0.821** ±0.003	0.473 ±0.001
***Average*** ***values***	*0.723* *±0.002*	***0.709*** *±0.003*	*0.769* *±0.003*	*0.597* *±0.002*	***0.822*** *±0.003*	*0.694* *±0.001*	***0.797*** *±0.002*	***0.620*** *±0.001*

On the Tomato-Village dataset (Table 4), TDD-YOLO clearly outperforms the baseline across all evaluation metrics, based on their average values. More particularly, with TDD-YOLO, the average precision is increased from 0.859 to 0.886 (+3.1%), the average recall is improved from 0.781 to 0.805 (+3.1%), the average mAP@50 is increased from 0.838 to 0.872 (+4.1%), and the average mAP@50:95 is improved from 0.546 to 0.579 (+6.0%). The improvement in precision indicates that the newly designed CAFE module enhances the feature extraction capability of the backbone and helps prevent the loss of low-level details. The increase in mAP@50 shows that more diseased instances are correctly detected by the proposed model, demonstrating that the newly designed CSAF modules, incorporated into it, improve its localization accuracy w.r.t. bounding boxes, thus reducing missed detections. The improvement in mAP@50:95 further suggests that TDD-YOLO is able to maintain high localization quality under stricter IoU thresholds, which is beneficial for more accurate estimation of diseased regions. These improvements demonstrate that introducing the CAFE and MSFF modules into the proposed model strengthens its ability to detect and localize diseases under complex field conditions.

Notably, the most pronounced detection improvements are observed w.r.t. Potassium Deficiency (with a mAP@50 increase of 10.4% and a mAP@50:95 increase of 11.6%) and Nitrogen Deficiency (with a mAP@50 increase of +7.0% and a mAP@50:95 increase of 9.4%). These types of diseases often manifest as subtle changes in color and texture rather than well-defined lesion contours. By combining the improved coordinate attention with asymmetric convolution, the novel CAFE module strengthens the extraction and fusion of fine spatial details along the horizontal and vertical directions, which helps the proposed model capture such small-scale lesions. Meanwhile, each MSFF module aggregates information across different receptive fields through a multi-branch structure, enabling the network to jointly model local lesion details and broader contextual cues, thereby jointly improving the model’s performance. This proves that compared to the baseline, the proposed TDD-YOLO model not only correctly identifies tomato diseases but also reduces missed and false detections, maintaining high detection performance while exhibiting stronger generalization ability. However, detecting Late Blight and Leaf Miner remains challenging for TDD-YOLO.

On the Tomato-Disease dataset (c.f., Table 5), TDD-YOLO likewise demonstrates better overall detection performance than YOLOv11n. For instance, the average precision increases from 0.723 to 0.822 (+13.7%), while mAP@50 and mAP@50:95 increase from 0.769 and 0.597 to 0.797 (+3.6%) and 0.620 (+3.9%), respectively. This indicates that, compared to the baseline, TDD-YOLO can produce more accurate predictions, yielding more reliable bounding boxes under different IoU thresholds and significantly reducing false positives. Although the average recall decreases slightly from 0.709 to 0.694 (−2.1%), this shows that the proposed model makes a certain trade-off between precision and recall, with fewer healthy leaves being misclassified as diseased. In practical disease management, reducing false alarms is particularly important for avoiding unnecessary control operations and economic losses; therefore, under comparable recall, achieving higher precision is generally more consistent with application needs.

The significant improvement in precision is mainly attributed to the introduction of the newly designed CSAF modules in the TDD-YOLO’s neck, which jointly model channel and spatial attention, effectively suppressing background interference and highlighting true lesion regions. At the category level, Septoria and Early Blight exhibit the most notable improvements in mAP@50, with an increase from 0.519 to 0.623 (+20.0%) and from 0.715 to 0.787 (+10.1%), respectively. This demonstrates that the CSAF modules can guide the proposed model to focus more on irregular diseases while suppressing similar background regions, thereby improving both its classification and localization performance. However, detecting Spider Mites and Yellow Leaf Curl Virus remains challenging.

**Fig 9:**
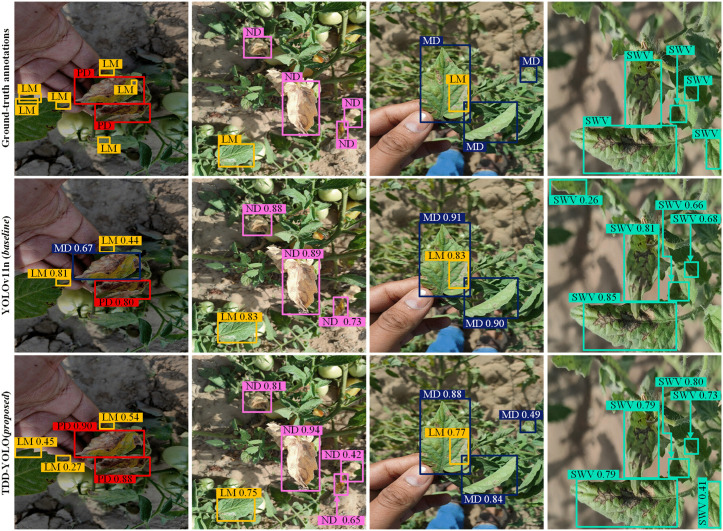
Sample visualizations of tomato-disease detections of the proposed TDD-YOLO and the baseline (YOLOv11n), performed on the Tomato-Village dataset w.r.t. Magnesium Deficiency (MD), Spotted Wilt Virus (SWV), Nitrogen Deficiency (ND), Leaf Miner (LM), and Potassium Deficiency (PD).

**Fig 10:**
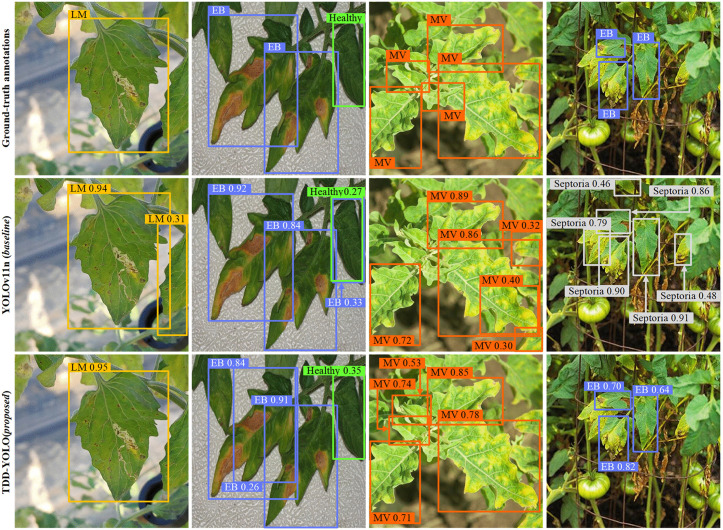
Sample visualizations of tomato-disease detections of the proposed TDD-YOLO model and the baseline (YOLOv11n), performed on the Tomato-Disease dataset w.r.t. Leaf Miner (LM), Early Blight (EB), and Mosaic Virus (MV).

Sample tomato-disease detection results of both compared models are visualized in Figs 9 and 10. Compared to the baseline (YOLOv11n), the proposed TDD-YOLO model not only better detects tomato diseases of various categories but also reduces instances of missed and false detections. This demonstrates that TDD-YOLO maintains high detection performance while also achieving enhanced robustness.

**Fig 11:**
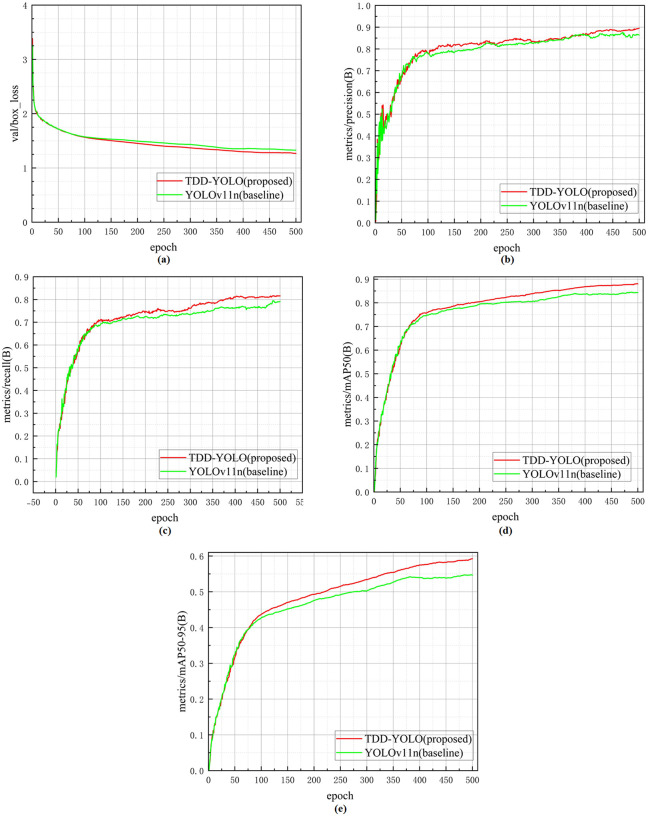
Comparison of regression performance and convergence speed of the proposed TDD-YOLO model and the baseline (YOLOv11n), performed on the Tomato-Village dataset: (a) validation box regression loss. (b) precision. (c) recall. (d) mAP@50. (e) mAP@50:95.

A comparison of the regression performance and convergence speed of the proposed TDD-YOLO model and the baseline, obtained on the Tomato-Village dataset, is shown in Fig 11. It can be observed in Fig 11a that the loss curve of TDD-YOLO (in red color) remains consistently lower than that of YOLOv11n (in green color), and eventually stabilizes. This indicates that TDD-YOLO is able to learn more effectively during training, reducing the training error. Additionally, TDD-YOLO demonstrates a rapid upward trend in detection precision (Fig 11b), recall (Fig 11c), mAP@50 (Fig 11d), and mAP@50:95 (Fig 11e), all of which are higher than those of YOLOv11n. This shows that TDD-YOLO not only converges more quickly but also maintains higher detection performance than the baseline, thereby validating its effectiveness and efficiency for application in complex agricultural scenarios.

**Fig 12:**
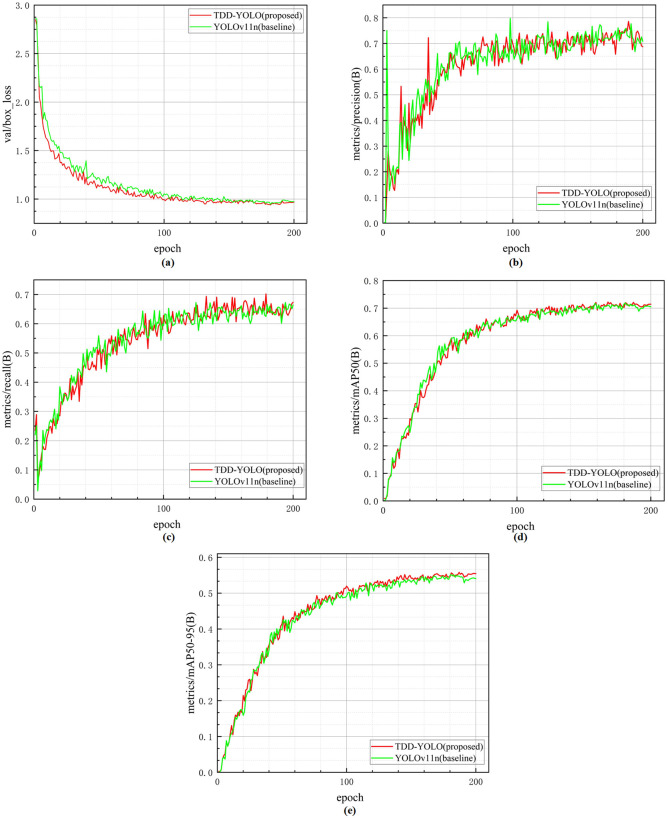
Comparison of regression performance and convergence speed of the proposed TDD-YOLO model and the baseline (YOLOv11n), performed on the Tomato-Disease dataset: (a) validation box regression loss; (b) precision; (c) recall; (d) mAP@50; (e) mAP@50:95.

On the Tomato-Village dataset, as shown in Fig 12a, the loss curve of TDD-YOLO (in red color) decreases rapidly and remains consistently lower than that of YOLOv11n (in green color), and eventually stabilizing, further confirming the faster convergence of TDD-YOLO. Although the precision, recall, and mAP@50 curves of YOLOv11n (Figs 12b, 12c, and 12d, respectively) show at some points faster increase than that of TDD-YOLO, they ultimately stabilize at values similar to that of TDD-YOLO. More importantly, TDD-YOLO reaches higher values of mAP@50:95 and mAP@50, compared to YOLOv11n, further proving that it is capable of accurately localizing disease regions under more stringent conditions, leading to better overall tomato-disease detection.

### Detection performance analysis

The experimental results, obtained on the Tomato-Village and Tomato-Disease datasets, demonstrate that the proposed TDD-YOLO model significantly improves detection performance compared to the baseline. To further display the practical performance of TDD-YOLO, we qualitatively compare its detection results with original annotated images of the Tomato-Disease dataset.

**Fig 13:**
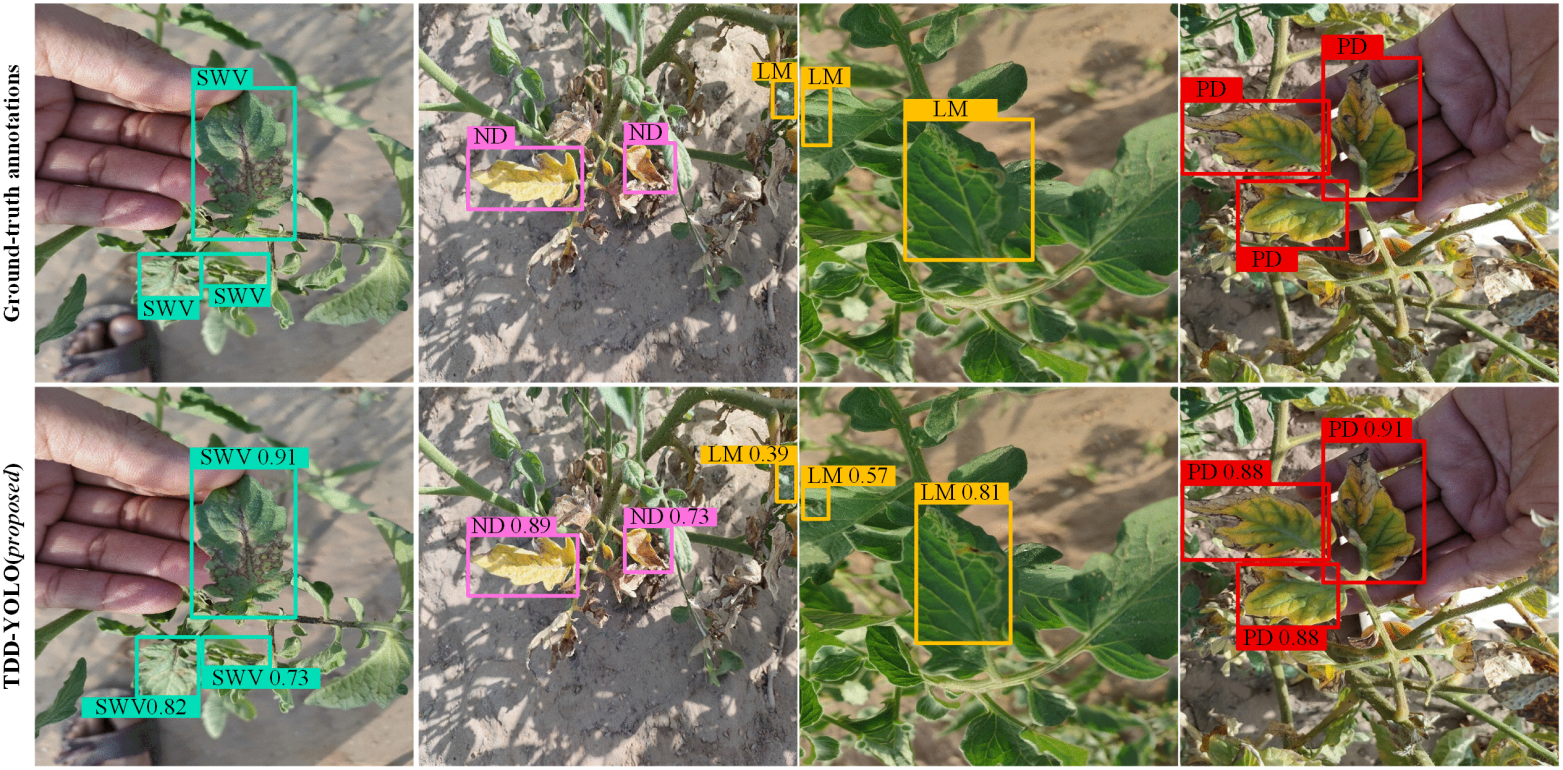
Representative samples of successful tomato-disease detections of the proposed TDD-YOLO model, performed on the Tomato-Disease dataset w.r.t. Spotted Wilt Virus (SWV), Nitrogen Deficiency (ND), Leaf Miner (LM), and Potassium Deficiency (PD).

Fig 13 illustrates representative samples of successful detections of tomato diseases, performed by the proposed TDD-YOLO model. As can be seen, TDD-YOLO is able to correctly identify tomato diseases at various scales by accurately localizing the disease regions. The detection results closely match the actual disease areas, demonstrating the proposed model’s effectiveness in recognizing and localizing different types of tomato diseases.

**Fig 14:**
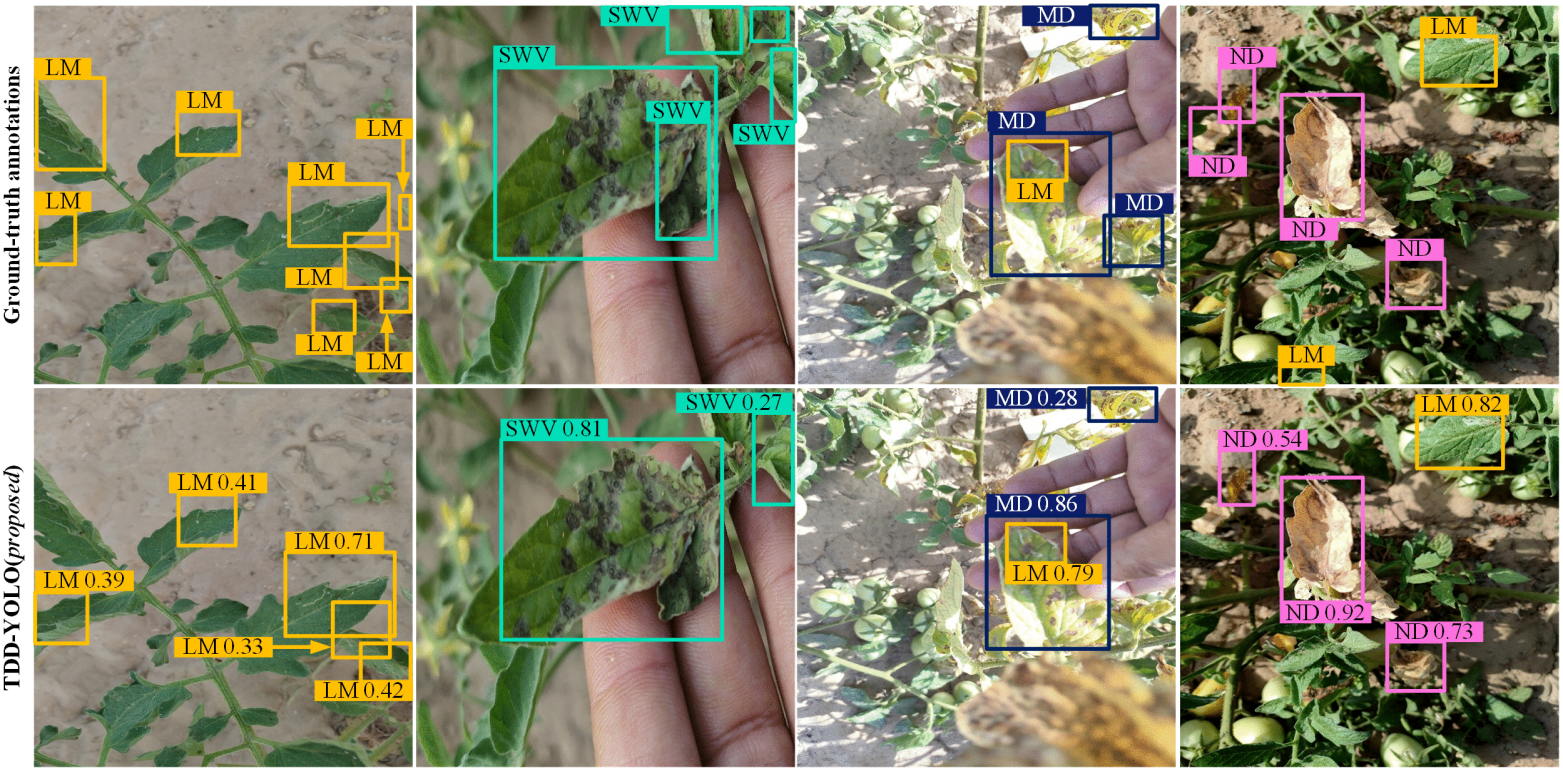
Representative samples of missed tomato-disease detections of the proposed TDD-YOLO model on the Tomato-Disease dataset w.r.t. Leaf Miner (LM), Spotted Wilt Virus (SWV), Magnesium Deficiency (MD), and Nitrogen Deficiency (ND).

Fig 14 illustrates representative samples of missed tomato-disease detections of TDD-YOLO on the Tomato-Disease dataset. As can be observed, when diseases are dense, the proposed model exhibits weaker recognition ability for smaller lesions, leading to missed detections in diseases such as Leaf Miner and Nitrogen Deficiency. The main reason for the missed detection of certain lesions is that early-stage lesions are small with limited pixel coverage, and some diseases are partially occluded by other stems and leaves, resulting in insufficient feature representation and limiting the proposed model’s effective detection capability in such cases.

From these results, one can observe the effectiveness of the proposed TDD-YOLO model in tomato-disease detection scenarios, while also revealing some performance bottlenecks, along with areas for improvement in complex scenarios. Future work will focus on further improving the training data and the structure of the model in order to enhance its ability to detect subtle lesions and reduce missed detections.

### Performance comparison with state-of-the-art models

Next, we compared the proposed TDD-YOLO model with state-of-the-art object detection models, including Faster R-CNN [26], Cascade R-CNN [27], YOLOv5n [28], YOLOv6n [29], YOLOv8n [30], YOLOv9t [31], YOLOv10n [32], and YOLOv11n [24]. The experimental results, obtained on the Tomato-Village and Tomato-Disease datasets, are shown in Tables 6 and 7, respectively.

**Table 6:** Performance comparison with state-of-the-art models, based on the Tomato-Village dataset (the best values are bolded).

Model	Average precision	Average recall	Average mAP@50	Average mAP@50:95	Parameter count (M)	FLOPs (G)	FPS
**Cascade** **R-CNN**	0.756 ±0.002	0.652 ±0.002	0.728 ±0.004	0.448 ±0.002	69.17	162.0	30.28
**Faster** **R-CNN**	0.739 ±0.002	0.626 ±0.002	0.696 ±0.003	0.380 ±0.002	41.36	134.0	38.34
**YOLOv5n**	0.847 ±0.001	0.782 ±0.002	0.845 ±0.002	0.534 ±0.001	2.50	7.1	64.61
**YOLOv6n**	0.817 ±0.006	0.690 ±0.002	0.772 ±0.004	0.497 ±0.003	4.23	11.8	**73.21**
**YOLOv8n**	0.859 ±0.005	0.742 ±0.004	0.815 ±0.002	0.513 ±0.002	3.00	8.1	68.29
**YOLOv9t**	0.839 ±0.006	0.741 ±0.005	0.815 ±0.001	0.502 ±0.003	**1.97**	7.6	38.33
**YOLOv10n**	0.856 ±0.005	0.768 ±0.004	0.838 ±0.004	0.553 ±0.003	2.70	8.2	67.52
**YOLOv11n** **(*baseline*)**	0.859 ±0.003	0.781 ±0.001	0.838 ±0.002	0.546 ±0.002	2.59	**6.3**	63.79
**TDD-YOLO** **(*proposed*)**	**0.886** ±0.003	**0.805** ±0.001	**0.872** ±0.002	**0.579** ±0.001	2.59	6.5	51.54

**Table 7:** Performance comparison with state-of-the-art models, based on the Tomato-Disease dataset (the best values are bolded).

Model	Average precision	Average recall	Average mAP@50	Average mAP@50:95	Parameter count (M)	FLOPs (G)	FPS
**Cascade** **R-CNN**	0.762 ±0.003	0.622 ±0.003	0.750 ±0.004	0.570 ±0.002	69.17	162.0	30.28
**Faster** **R-CNN**	0.684 ±0.004	0.697 ±0.003	0.773 ±0.003	0.535 ±0.001	41.36	134.0	38.34
**YOLOv5n**	0.747 ±0.003	0.675 ±0.004	0.757 ±0.003	0.573 ±0.002	2.50	7.1	64.61
**YOLOv6n**	0.746 ±0.002	0.686 ±0.004	0.763 ±0.002	0.601 ±0.001	4.23	11.8	**73.21**
**YOLOv8n**	0.781 ±0.005	0.677 ±0.002	0.778 ±0.003	0.601 ±0.001	3.00	8.1	68.29
**YOLOv9t**	0.771 ±0.002	0.683 ±0.003	0.769 ±0.002	0.607 ±0.001	**1.97**	7.6	38.33
**YOLOv10n**	0.771 ±0.002	0.682 ±0.005	0.736 ±0.003	0.571 ±0.002	2.70	8.2	67.52
**YOLOv11n** **(*baseline*)**	0.723 ±0.002	**0.709** ±0.003	0.769 ±0.003	0.597 ±0.002	2.59	**6.3**	63.79
**TDD-YOLO** **(*proposed*)**	**0.822** ±0.003	0.694 ±0.001	**0.797** ±0.002	**0.620** ±0.001	2.59	6.5	51.54

The results in Tables 6 and 7 clearly show that the proposed TDD-YOLO model outperforms all state-of-the-art models on both datasets according to all evaluation metrics, except for average recall on the Tomato-Disease dataset where the winner is the baseline (YOLOv11n).

On the Tomato-Village dataset (c.f., Table 6), compared with two-stage models (Cascade R-CNN and Faster R-CNN), TDD-YOLO has clear advantages not only w.r.t. detection performance but also the model size and FLOPs, making these models difficult to deploy on edge devices such as unmanned aerial vehicles (UAVs) or mobile inspection robots utilized for real-time detection. Among one-stage models, although YOLOv9t exhibits the lowest parameter count (1.97 M), all the remaining evaluation metric values are worse compared to the proposed TDD-YOLO model. Compared with the baseline (YOLOv11n), TDD-YOLO increases the average precision by 3.1%, indicating that the newly designed CAFE module can effectively strengthen the extraction of disease features, while the average recall increases also by 3.1%, showing that the proposed model is able to better detect small and occluded lesions. At the same time, FPS of TDD-YOLO reached 51.54, exceeding the benchmark threshold of 30 FPS for real-time detection, indicating that the proposed model has good inference efficiency while maintaining high detection performance, making it suitable for deployment on resource limited edge devices.

Experimental results in Table 7, obtained on the Tomato-Disease dataset, demonstrate that the proposed TDD-YOLO model is also superior according to 3 (out of 4) evaluation metrics. Only based on average recall TDD-YOLO takes second place with a value of 0.694, which is slightly lower than that of YOLOv11n (0.709). The TDD-YOLO’s average precision (0.822) is 13.7% higher than that of YOLOv11n (0.723), while also improving average mAP@50 (to 0.797) and average mAP@50:95 (to 0.620) by 3.6% and 3.9%, respectively, thus fully verifying the synergistic effect of feature enhancement and channel-spatial attention in improving disease feature representation and boundary localization accuracy.

**Fig 15:**
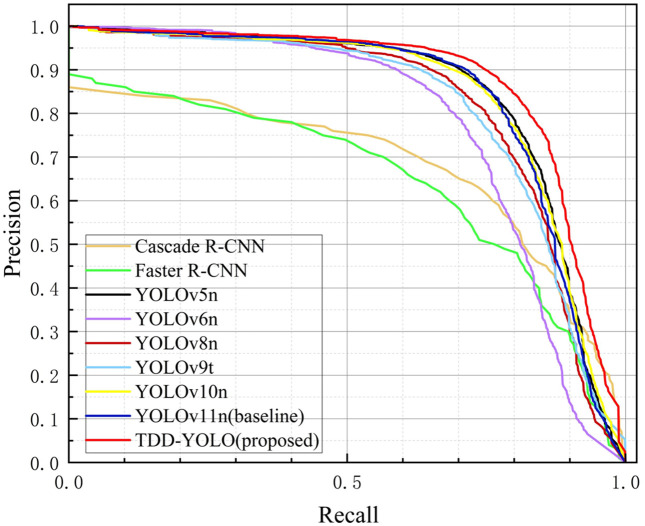
Macro-averaged precision–recall curves of models, compared on the Tomato-Village dataset.

**Fig 16:**
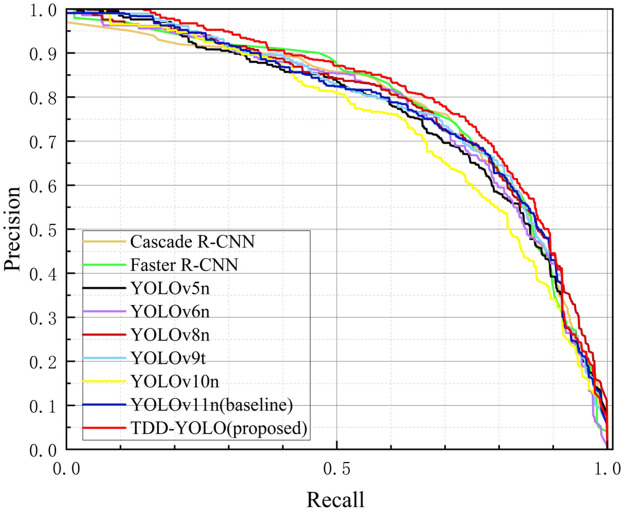
Macro-averaged precision–recall curves of models, compared on the Tomato-Disease dataset.

Figs 15 and 16 depict precision–recall curves of models, compared on the Tomato-Village and Tomato-Disease datasets, respectively. Analysis of these figures indicates that the proposed TDD-YOLO model has the largest Area Under the Curve (AUC), which is proof of its superior tomato-disease detection performance in comparison to state-of-the-art models on both datasets.

### Ablation study

To verify the impact of the newly designed modules (MSFF, CSAF, CAFE) on the performance of the proposed model and investigate their interactions, we conducted an ablation study using the Tomato-Village dataset. The obtained results are shown in Table 8.

Step 0 involved only the pure baseline (YOLOv11n) without any extra modules added to it. In steps 1–3, the newly designed module types were individually added to the baseline. This led to improving all evaluation metrics, except for recall when using the MSFF component solely (Step 1). This configuration allowed the model to reduce its parameter count from 2.59 M to 2.17 M (−16.2%) and FLOPs from 6.3 G to 6.1 G (−3.2%), while at the same time increasing average precision, mAP@50, and mAP@50:95 compared to the baseline. Even though the FPS decreased from 63.79 to 58.47, it still reflects a good balance between efficiency and performance. This demonstrates that MSFF enlarges the effective receptive field through multi-scale feature fusion, preserves disease features across different scales, and improves localization performance. When incorporating the CSAF module type alone, the synergistic enhancement of feature representation and attention mechanisms enabled the resultant model to improve all evaluation metrics, compared to YOLOv11n, indicating that it is able to maintain high localization accuracy even under stricter IoU requirements, thereby strengthening its bounding-box regression capability. However, the CSAF addition also raised the model’s parameter count to 2.68 M (+3.5%) and FLOPs to 6.5 G (+3.2%), while FPS dropped from 63.79 to 35.47, indicating that inference speed is sacrificed to some extent when improving the performance. The addition of the CAFE module (Step 3) had the most significant effect on improving the detection performance across all evaluation metrics, demonstrating that CAFE effectively enhances the model’s feature extraction capability. Particularly, compared to the baseline, average precision was increased from 0.859 to 0.879 (+2.3%), average recall improved from 0.782 to 0.791 (+1.2%), average mAP@50 boosted from 0.838 to 0.855 (+2.0%), and average mAP@50:95 increased from 0.546 to 0.564 (+3.3%), while maintaining a relatively high inference speed (60.23 FPS). However, adding CAFE to the baseline also raised the parameter count from 2.59 M to 2.90 M and FLOPs from 6.3 G to 6.6 G.

In steps 4–6, the newly designed module types were added to the baseline in pairs. In the ‘MSFF + CSAF’ paired combination (Step 4), the MSFF component captured richer disease-related information by fusing features from multiple hierarchical levels, thereby enabling CSAF to better discriminate between different disease types and enhancing the model’s localization capability, although FPS decreased to 53.41. In the ‘MSFF + CAFE’ pair (Step 5), the CAFE component enhanced the model’s ability to extract local features by enlarging the effective receptive field, enabling it to preserve richer disease-related characteristics; subsequently, the MSFF component fused these features through its multi-branch architecture to integrate diverse multi-scale representations, allowing the model to more accurately localize and distinguish between different types of diseases, allowing it to achieve the so-far highest average recall (0.803) and average mAP@50 (0.859), while maintaining a relatively high inference speed (57.43 FPS). In the ‘CSAF + CAFE’ combination (Step 6), the richer disease representations extracted by CAFE empower CSAF to capture more comprehensive contextual features, which jointly improved the model’s capacity to differentiate diseases and localize lesions, enabling it to achieve the best mAP@50:95 so far (0.570). However, FPS dropped to 32.87, highlighting the negative impact of CSAF on inference speed.

In the final step 7, all three module types were incorporated together into the baseline, resulting in the proposed TDD-YOLO model. This configuration achieved the best results across all evaluation metrics, confirming that the newly designed modules are most effective when combined. Meanwhile, the parameter count remained at 2.59 M (as in the baseline), while FLOPs were kept at 6.5 G – an increase of only 0.2 G. Although FPS decreased to 51.54, the model still satisfied the real-time performance requirement of 30 FPS. This represents a performance gain achieved with only a slight increase in computational cost, confirming the complementary nature of feature enhancement, attention modeling, and multi-scale feature fusion. These results demonstrate that the proposed modules collectively enhance fine-grained feature extraction, strengthen localization precision, and reduce computational overhead, ultimately providing a high-accuracy and high-efficiency framework for practical tomato-disease detection.

Although the newly designed MSFF, CSAF, and CAFE modules, utilized by the proposed TDD-YOLO model, replace heavier modules in the baseline, parameter count remains unchanged, which is attributed to the additional structural complexity of TDD-YOLO that reduces the effect of the lightweight module design. Specifically, the incorporation of CAFE and CSAF modules into the baseline increases parameter count to 3.00 M and FLOPs to 6.7 G. To enable deployment on resource-constrained devices, the proposed model replaces the C3k2 modules in the neck with lightweight MSFF modules. Through an efficient multi-branch feature fusion mechanism, each MSFF module improves detection performance while reducing computational overhead. As shown in the MSFF-only setting (Step 1), introducing the MSFF component alone reduces parameter count to 2.17 M and FLOPs to 6.1 G, effectively compensating for the increases in parameter count and FLOPs brought by CAFE and CSAF modules. This way, TDD-YOLO not only achieves better detection performance but also maintains a good balance between accuracy and computational efficiency, making it suitable for deployment in resource-limited environments.

**Fig 17:**
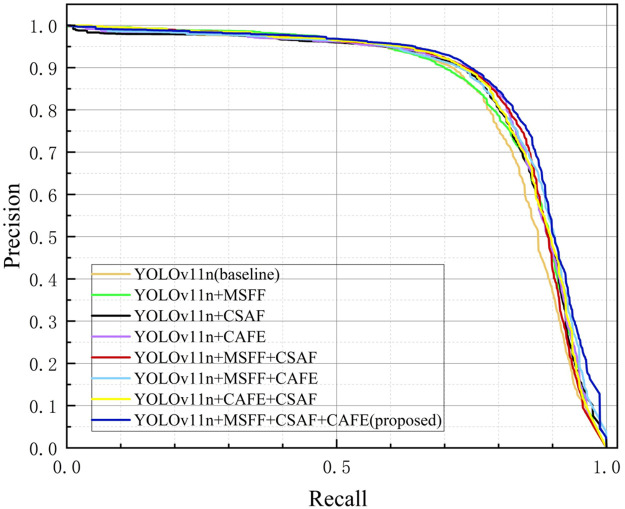
Macro-averaged precision–recall curves, obtained in the ablation study.

Precision–recall curves of the ablation study experiments are visualized in Fig 17. There, one can see that the AUC of the proposed TDD-YOLO model, obtained in step 7 (‘YOLOv11n + MSFF + CSAF + CAFE’), was significantly greater than that obtained in all other steps, demonstrating that the model (TDD-YOLO), produced in the final step, achieved indeed the best tomato-disease detection performance, compared to all previous steps of the ablation study.

### Performance comparison of attention mechanisms

In previous experiments, it was demonstrated that the newly proposed CSAF mechanism allows the proposed model to effectively capture both channel and spatial information. It also learns complex dependencies between different dimensions, thereby enabling the proposed model to focus better on disease regions. To further evaluate the performance of CSAF to that of other attention mechanisms, we conducted experiments with the baseline (YOLOv11n) on the Tomato-Village dataset by first incorporating the newly designed CSAF component into it, and then consequently replacing it with the CBAM [15], the Large Selective Kernel network (LSK) module [33], and the Efficient Channel Attention (ECA) module [34] for comparison. Each of these attention module types was incorporated into the baseline exactly at the same positions as the CSAF module type. The experimental results are shown in Table 9.

**Table 9:** Performance comparison of different attention modules, utilized by the baseline (YOLOv11n) for object detection based on the Tomato-Village dataset (the best values are bolded).

Combination	Average Precision	Average Recall	Average mAP@50	Average mAP@50:95	Parameter count (M)	FLOPs (G)	FPS
**Baseline + CBAM**	**0.880** ±0.002	0.768 ±0.002	0.844 ±0.002	0.544 ±0.001	2.68	6.5	47.03
**Baseline + LSK**	0.872 ±0.002	0.752 ±0.003	0.836 ±0.002	**0.559** ±0.002	3.28	9.0	**52.83**
**Baseline + ECA**	0.866 ±0.002	0.778 ±0.005	0.845 ±0.003	0.550 ±0.001	**2.58**	**6.3**	51.12
**Baseline + CSAF**	0.875 ±0.003	**0.790** ±0.002	**0.851** ±0.002	0.553 ±0.001	2.68	6.5	35.47

According to Table 9, CSAF outperforms all other attention modules in terms of average recall and average mAP@50, while also being the first runner-up on the other two metrics (i.e., average precision and average mAP@50:95, respectively after CBAM and LSK). Although ECA has the smallest number of parameters and FLOPs, all its results are lower than those of CSAF. Furthermore, parameter count of the ‘Baseline + CSAF’ combination is only 0.1 M greater than that of the ‘Baseline + ECA’ combination, while its FLOPs value is higher by just 0.2 G, demonstrating that the incorporation of the CSAF module type into the baseline can enhance the ability of the model to focus better on tomato-disease features and improve its detection performance, while maintaining a reasonable computational complexity. Although the FPS value of ‘Baseline + CSAF’ is relatively low (35.47), it still maintains a reasonable computational efficiency, making it suitable for deployment in resource constrained environments, as it remains above the real-time detection threshold of 30 FPS and thus can satisfy the deployment requirements of most edge devices in resource-limited agricultural scenarios. Since tomato-disease detection prioritizes accurate disease identification, we adopted CSAF to enhance attention to lesion regions and reduce missed detections under acceptable constraints of computational complexity and inference speed, reflecting the trade-off between detection performance and speed.

Further, we employed the Gradient-weighted Class Activation Mapping (Grad-CAM) to visualize the regions of interest (RoIs) of the baseline model (YOLOv11n) in tomato-disease images, thereby enhancing the interpretability of detection results [35]. Grad-CAM calculates the gradients of the target category with respect to each feature map and performs a weighted summing across all channels to generate a visual explanation, highlighting the regions in the images where the model focused, thus allowing verification of whether the model truly captures relevant features. The method can be mathematically expressed as follows:


$$\begin{array}{c} \overset{c}{\underset{Grad-CAM}{L}}=ReLU\left(\sum_{k}\overset{c}{\underset{k}{\alpha }}A^{k}\right) \end{array}$$ (15)

where $\overset{c}{\underset{Grad-CAM}{L}}$ denotes the final heatmap generated by computing the weighted sum of the overall activation values of the k feature map and the corresponding importance weights $\overset{c}{\underset{k}{\alpha }}$, followed by a ReLU activation function to produce a nonlinear map. The importance weight of the k feature map for category c is obtained as follows:


$$\begin{array}{c} \overset{c}{\underset{k}{\alpha }}=\frac{\text{1}}{Z}\sum_{i}\sum_{j}\frac{{\partial y}_{c}}{\overset{k}{\underset{ij}{\partial A}}} \end{array}$$ (16)

where $y_{c}$ denotes the predicted probability of the target category c, $\overset{k}{\underset{ij}{A}}$ denotes the activation value at position (i,j) of the k feature map in the convolutional layer, and Z denotes the normalization factor used to ensure that the computed gradient reflects the average value.

**Fig 18:**
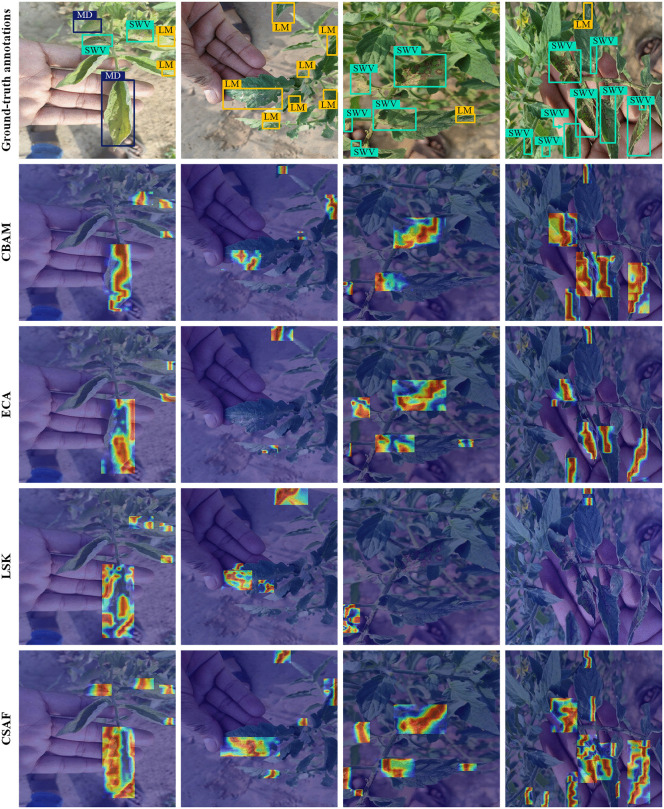
Grad-CAM generated heatmaps, visualizing RoIs with different attention mechanisms used by the baseline (YOLOv11n) w.r.t. Magnesium Deficiency (MD), Leaf Miner (LM), and Spotted Wilt Virus (SWV).

We performed backpropagation on the 21^st^ and 25^th^ layers of YOLOv11n and used Grad-CAM to generate heatmaps that visualized RoIs under different attention mechanisms, as shown in Fig 18. There, regions with darker red color indicate the highest attention weights assigned by the baseline (YOLOv11n), followed by the yellow color. Blue and green areas correspond to lower attention levels. Among different attention mechanisms compared, the newly designed CSAF shows more extensive red distribution over the diseased regions, meaning that it is more effective in distinguishing disease-affected areas from the background and in highlighting key disease features. In addition, CSAF demonstrates better performance in detecting small lesions, which is a prerequisite for reducing both the miss rate and false positive rate under complex agricultural conditions.

## Discussion

This paper proposes a novel TDD-YOLO model, which builds upon YOLOv11n and effectively addresses the challenges present in tomato-disease detection by incorporating feature enhancement, channel-spatial attention fusion, and multi-scale feature fusion. Experimental results, obtained on two tomato-disease datasets, demonstrate that TDD-YOLO consistently outperforms the baseline, along with state-of-the-art models, while maintaining the same number of parameters with only a slight increase in FLOPs. The proposed model achieves superior performance on key evaluation metrics, such as precision, recall, mAP@50, and mAP@50:95, maintaining a high detection performance under complex field conditions, which is particularly important for deployment in resource-constrained agricultural environments.

First, the complexity of field environments—including variable lighting conditions, high diversity among disease types (with significant visual differences across categories), and cluttered backgrounds (e.g., shadows and occlusions)—poses numerous challenges for disease detection. To address these challenges, we first designed a CAFE module for utilization by the proposed model, which integrates asymmetric convolution with local contextual information. By strengthening fine-grained spatial cues along both horizontal and vertical directions, this module effectively enhances the backbone network’s sensitivity to small-scale and subtle disease symptoms, thereby facilitating the capture of common field-image characteristics such as blurred lesion boundaries, minor color variations, and illumination changes.

Second, the newly designed CSAF module type explicitly models channel-wise and spatial-wise dependencies through parallel attention branches, and employs a fusion mechanism to preserve their nonlinear interactions, enabling the proposed model to more effectively suppress background interference from regions such as soil and shadows—a capability that is particularly critical for reducing both false positives and missed detections in complex scenarios.

The third newly designed module type (MSFF) adopts a multi-branch architecture to fuse features across different scales, allowing the proposed model to simultaneously capture local lesion details and high-level semantic context, thus exhibiting stronger high-level semantic robustness when disease lesions vary significantly in scale.

Despite achieving high accuracy, the proposed TDD-YOLO model may still produce false positives or miss low-contrast lesions when distinguishing diseases in high-density infection scenarios or under extreme lighting conditions—such as strong backlighting, severe shadows, or specular reflections. Additionally, the disease categories in the datasets used in this study exhibit imbalanced distributions, which adversely affects the proposed model’s generalization ability for detecting rare diseases. Although TDD-YOLO still overall outperforms the baseline and other state-of-the-art models under these challenging conditions, its relative performance advantage is somewhat reduced, indicating that both intense illumination variations and data imbalance negatively impact all detectors to some extent, and that further improvements in model robustness remain necessary.

Future work will prioritize two main directions. First, we are going to construct a more diverse and category-balanced tomato-disease dataset, incorporating samples collected from different regions, time periods, cultivars, and growth stages, as well as specifically curated images capturing extreme lighting and occlusion scenarios. By integrating techniques such as domain adaptation, targeted data augmentation, and active learning, the model’s generalization ability in unseen environments and for rare diseases is expected to improve. Second, we will further explore lightweight variants of TDD-YOLO through methods such as channel pruning and knowledge distillation, aiming to reduce both parameter count and computational overhead while maintaining high detection performance as much as possible. The optimized model will be deployed on embedded or edge devices and evaluated through field trials in real-world agricultural settings, with systematic assessment of latency, throughput, and usability feedback from agricultural experts and farmers. This step is crucial for translating the research outcomes into reliable tools for precision agriculture.

## Conclusion

The proposed TDD-YOLO model integrates local context enhancement, channel-spatial attention, and feature fusion to achieve a high-performance detection of tomato diseases, making significant progress in advancing precision agriculture and its intelligent development. This study suggests a way for significantly improving detection robustness in complex scenarios while maintaining low computational overhead, and for substantially improved disease detection, thus providing a feasible pathway for edge deployment of the proposed model. It lays the technical foundation for constructing early warning systems, reducing dependency on pesticide usage, and achieving early disease detection.

Despite the promising results, this study still has several limitations. First, due to the limited coverage of existing datasets, the generalization ability of the proposed model under unseen diseases, different tomato growth stages, and acquisition conditions from different regions remains to be further validated. Second, extreme illumination variations, severe occlusions (e.g., overlapping leaves), and harsh weather conditions (e.g., rain and dust) may still challenge its detection performance. Finally, although the proposed model incorporates lightweight components, further design improvements may be required for deployment on edge devices with strict latency requirements and memory constraints.

Future research will focus on building a diverse and sufficiently large agricultural disease dataset to enhance the generalization ability of the proposed model. Also, more targeted data augmentation strategies will be adopted to improve its robustness to illumination and occlusion variations. In addition, distillation and compression techniques will be explored to enable model deployment on edge and embedded devices, further promoting the application of intelligent disease detection technologies in crop protection practices.
